# Exploring the Link Between Psoriasis and Adipose Tissue: One Amplifies the Other

**DOI:** 10.3390/ijms252413435

**Published:** 2024-12-15

**Authors:** Roberta Di Caprio, Ersilia Nigro, Eugenia Veronica Di Brizzi, Dario Buononato, Marta Mallardo, Vittorio Tancredi, Aurora Daniele, Anna Balato

**Affiliations:** 1 Unit of Dermatology, University of Campania Luigi Vanvitelli, 80138 Naples, Italy; 2 Department of Molecular and Biotechnological Medicine, University of Naples “Federico II”, 80131 Naples, Italy; 3 CEINGE Franco Salvatore Advanced Biotechnology, 80145 Naples, Italy; 4 Department of Environmental, Biological and Pharmaceutical Sciences and Technologies, University of Campania Luigi Vanvitelli, 81100 Caserta, Italy

**Keywords:** psoriasis, obesity, adipose tissue, inflammation

## Abstract

Psoriasis and obesity, while distinct, are inter-related inflammatory conditions. Adipose tissue (AT)-derived mediators could be pathogenically active in triggering and/or amplifying psoriatic skin inflammation and, vice versa, skin inflammation could drive increased adiposity that triggers the development of several chronic conditions. Gaining insight into their intricate relationship could be essential for effective management and treatment. The aim of this study was to determine (i) the pathogenic role of psoriasis-signature cytokines in contributing to AT metabolism and (ii) the role of AT-derived mediators in triggering and/or amplifying skin inflammation. For this reason, firstly, whole AT was treated with IL-17 and TNF-α, alone or in combination, to investigate their effects on the expression and production of adipokines and inflammatory factors. IL-17 and TNF-α were able to induce an additive or synergistic effect on AT-derived mediators. In order to assess the effects on the skin of inflamed AT by psoriasis-signature cytokines, ex vivo skin organ culture was performed and an increase in several inflammatory mediators was observed. These findings confirm that psoriasis and obesity amplify each other’s inflammatory processes and understanding this mutual exacerbation could lead to more effective therapeutic strategies that address both skin inflammation and AT metabolism.

## 1. Introduction

Psoriasis is a chronic inflammatory skin disease associated with several comorbidities, including metabolic disorders such as obesity, type II diabetes, dyslipidemia, nonalcoholic fatty liver disease and metabolic syndrome (MetS) [1,2]. In particular, psoriasis and obesity, while distinct, are inter-related conditions with complex pathophysiological links. They often create a vicious cycle, each worsening the other’s impact. A study conducted by Snekvik et al. evaluated how factors such as body mass index (BMI), waist circumference, waist-to-hip ratio and changes in weight over a 10-year period could influence the risk of developing psoriasis. The findings revealed that individuals with obesity had a 1.87-fold higher risk of developing the condition, while those who gained 10 kg or more during the observed period had a 1.72-fold greater risk compared to those who maintained a stable weight [3]. However, although the precise mechanisms underlying the relationship between obesity and psoriasis are not yet fully understood, it is believed that systemic inflammation driven by adipose tissue (AT), especially visceral fat, plays a significant role contributing to psoriasis onset and exacerbation [4,5]. AT, especially when accumulated around internal organs, releases pro-inflammatory cytokines and other inflammatory mediators which might play a pivotal role in the onset and progression of cutaneous inflammation. Indeed, once considered an inert location of energy storage, studies over the last decade have identified AT as a major secretory organ which plays a central role in a complex network of endocrine and autocrine/paracrine crosstalk between organs and tissues such as heart, vasculature, liver, muscle, pancreas and skin tissues [6,7]. Uncontrolled adipocyte hypertrophy leads to abnormal adipokine release, in which there is an increase in pro-inflammatory adipokines (leptin, visfatin) and a reduction in anti-inflammatory adipokines (adiponectin), leading to local inflammation and immune cell recruitment [8]. Higher levels of inflammatory cytokines/chemokines are also secreted from the AT of obese individuals, contributing to the development of systemic inflammation. In addition to adipocytes, AT contains endothelial cells, fibroblasts, macrophages, myeloid cells and T helper (Th) cells [9]. In a lean state, Th2 cells, regulatory T cells (Tregs) and invariant Natural Killer T (NKT) cells are the dominant immune cell populations, whereas increased adiposity positively correlates with a marked infiltration of pro-inflammatory cells, including Th1 and Th17 cells [10]. This infiltration causes an enhanced expression of inflammatory cytokines that are also central in Pso pathogenesis, such as interleukin (IL)-17, IL-22, and interferon (IFN)-γ. For this reason, some authors underline that the link between AT inflammation and psoriasis is bidirectional, since AT-derived mediators could be pathogenically active in triggering and/or amplifying psoriatic skin inflammation and, vice versa, skin inflammation could drive increased adiposity that triggers the development of several chronic conditions. However, despite the wide array of research produced on the topic, clear characterization of these effects is still lacking. The aim of this study was to determine (i) the pathogenic role of psoriasis-signature cytokines in contributing to AT inflammation and (ii) the role of AT-derived mediators in triggering and/or amplifying skin inflammation.

## 2. Results

Skin specimens and subcutaneous AT fragments were obtained from healthy normal to overweight women, and there was no significant difference in the results based on weight.

### 2.1. AT Protein Array

To evaluate whether psoriasis-signature cytokines were able to modulate AT response, a protein array was performed in supernatants of AT stimulated with IL-17 and TNF-α. The components of this broad secretory profile include a large spectrum of proteins that can be organized into (i) adipocytokines, (ii) angiogenesis, (iii) immunity, (iv) migration and (v) glucose homeostasis.

**Figure 1:**
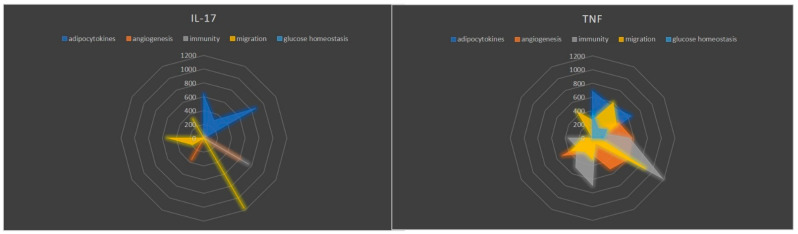
Radar plots comparing the effects of IL-17 and TNF-α on AT secretory profiles. Five categories of proteins are represented: adipocytokines (blue), angiogenesis-related factors (orange), immunity-related mediators (gray), migration-associated proteins (yellow) and glucose homeostasis regulators (light blue). IL-17 induced a moderate increase in adipocytokines and glucose homeostasis proteins, while TNF-α robustly enhanced proteins across all categories, particularly those involved in immunity and migration.

Adipocytokines (adiponectin, adipsin, leptin, LEPR and resistin) regulate metabolic and inflammatory processes. Angiogenesis-related proteins (ANGPT1, ANGPT2, PAI-1, PDGF-AA, PDGF-AB, PDGF-BB, TIMP-1, TIMP-2 and VEGF-A) are key for vascular remodeling and endothelial function. Immune mediators (IL-10, IL-11, IL12p70, IL-1R4, IL-6, IL-6R, IL-8, RANTES, SAA and TECK) contribute to inflammation and immune regulation. Migration-associated proteins (ENA-78, IP-10, lymphotactin, MCP-1, MCP-3, MIF, MIP-1β, MSP, SDF-1α and XEDAR) facilitate cell recruitment and tissue repair. Glucose homeostasis regulators (ANGPTL4, IGFBP-1, IGFBP-3 and insulin) maintain energy balance and metabolic control. As illustrated in Figure 1, TNF-α markedly enhanced the levels of all analyzed proteins compared to IL-17, which, in contrast, led to a moderate increase in adipocytokines.

### 2.2. ELISA

**Figure 2:**
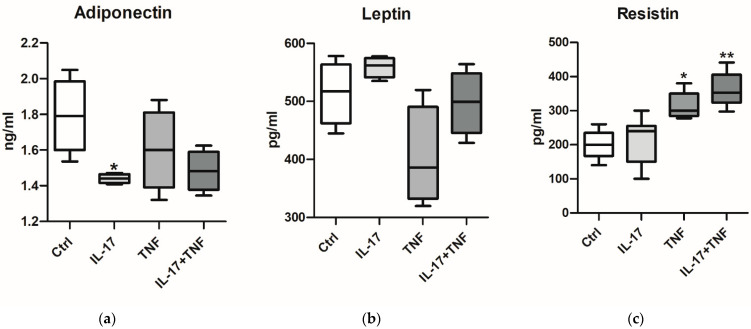
Protein levels of adiponectin (**a**), leptin (**b**) and resistin (**c**) in AT supernatants after stimulation with IL-17, TNF-α or their combination. Data are expressed as mean ± standard deviation (SD); statistical significance is indicated for comparisons between conditions (* *p* < 0.05; ** *p* < 0.01).

Taking into account the protein array results on adipocytokines, supernatants were analyzed, by using an ELISA, for the release of adiponectin, leptin and resistin (Figure 2). Specifically, adiponectin levels were downregulated by both IL-17 and TNF-α, although statistical significance was observed only with IL-17 treatment (Figure 2a). For leptin, no statistically significant differences were detected; however, a slight increase was noted following IL-17 stimulation (Figure 2b). Lastly, resistin levels were significantly elevated in response to TNF-α, either as a single treatment or in combination with IL-17 (Figure 2c).

### 2.3. Gene Expression

**Figure 3:**
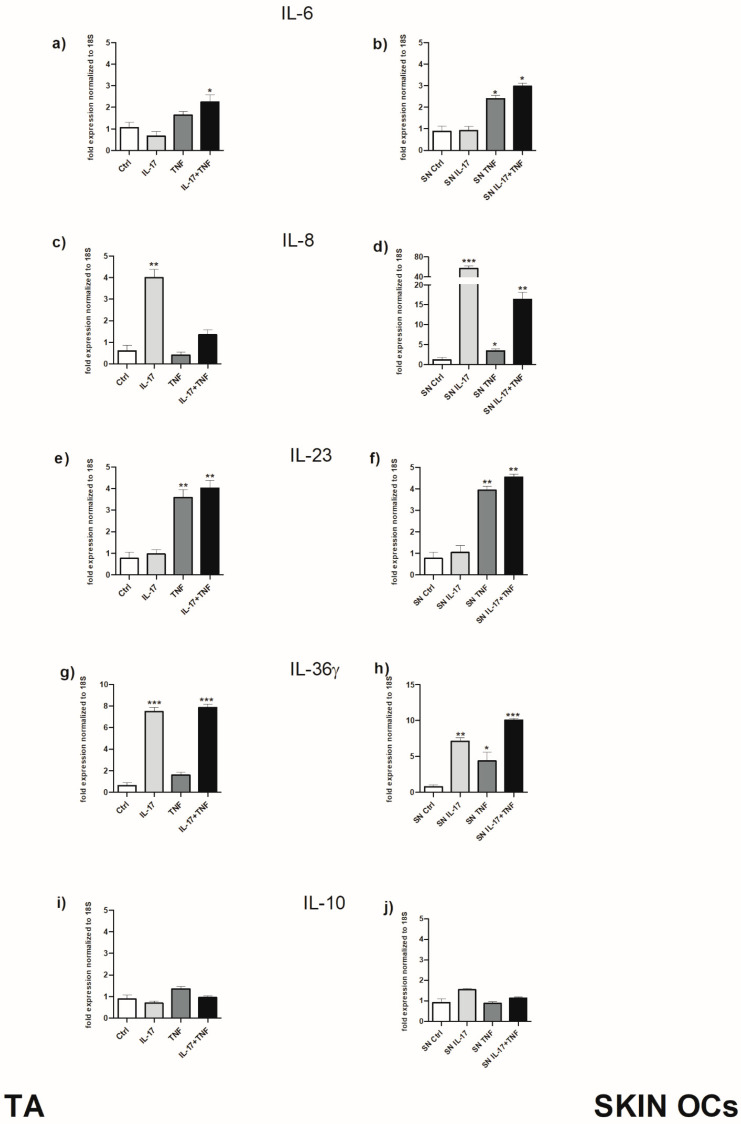
Effect of IL-17 and TNF-α on cytokine gene expression in AT fragments and corresponding skin OCs. Panels (**a**,**c**,**e**,**g**,**i**) show gene expression in AT stimulated with IL-17, TNF-α or their combination, while panels (**b**,**d**,**f**,**h**,**j**) depict cytokine expression in skin OCs treated with supernatants derived from stimulated AT. Cytokines analyzed include IL-6 (**a**,**b**), IL-8 (**c**,**d**), IL-23 (**e**,**f**), IL-36γ (**g**,**h**) and IL-10 (**i**,**j**). Statistical analysis was performed with respect to controls. Data are displayed as mean ± SD of triplicates pooled from three independent experiments. Mann–Whitney test was used to calculate significant differences. * *p* < 0.05; ** *p* < 0.01; *** *p* < 0.001.

To assess if the main psoriasis-signature cytokines could contribute to AT-driven inflammation, the gene expression of IL-6, IL-8, IL-23, IL-36γ and IL-10 was evaluated in AT fragments stimulated with IL-17, TNF-α or their combination. Moreover, to explore AT-promoting skin inflammation, the gene expression of the same cytokines was also determined in healthy skin OCs treated with AT-derived supernatants. IL-6 was significantly increased in AT stimulated with the combination of IL-17 and TNF-α (Figure 3a). Furthermore, in skin OCs treated with AT-derived supernatants, IL-6 levels were significantly elevated not only by the combination but also by TNF-α alone (Figure 3b), highlighting the important contribution of AT stimulated with TNF-α in driving skin inflammation. IL-8 was strongly upregulated by IL-17 in AT (Figure 3c). Interestingly, in skin samples, exposure to supernatants from AT stimulated with IL-17, TNF-α or their combination also led to a significant increase in IL-8 levels. This further underscores the pivotal role of TNF-α produced by stimulated AT in amplifying inflammation of the skin, particularly in synergy with IL-17 (Figure 3d). A similar trend was observed for IL-23 (Figure 3e,f) and IL-36γ (Figure 3g,h), although the contribution of TNF-α was slightly weaker in the case of IL-23. In contrast, IL-10 expression did not show any significant changes in either adipose tissue or skin under any of the tested conditions (Figure 3i,j). This suggests that IL-10 has a limited role in modulating inflammatory responses in this context.

## 3. Discussion

The findings from this study underscore the bidirectional inflammatory relationship between AT and psoriasis, providing critical insights into the mechanisms by which psoriasis-signature cytokines and AT-derived mediators influence one another. In particular, this study highlights that IL-17 and TNF-α, two key cytokines in psoriasis pathogenesis, significantly alter AT secretory profiles and gene expression, promoting a pro-inflammatory state that impacts skin homeostasis. Adipocytokines, key mediators linking metabolism and inflammation, were particularly affected by cytokine stimulation, reflecting their potential role in amplifying systemic and local inflammation. Moreover, protein array analysis revealed that TNF-α markedly enhanced adipocytokine levels, while IL-17 showed more moderate effects. This pattern was confirmed by the ELISA, where adiponectin levels were significantly reduced by IL-17 and, to a lesser extent, by TNF-α. As adiponectin is known to exert anti-inflammatory effects and inhibit IL-17 production, its downregulation may create a permissive environment for sustained inflammation [11]. Conversely, resistin, a pro-inflammatory adipocytokine associated with Th1/Th17 immune responses, was significantly elevated by TNF-α alone or in combination with IL-17, supporting its role in exacerbating inflammatory pathways relevant to psoriasis [12]. Leptin showed only slight increases with IL-17, consistent with its systemic role in inflammation, although it did not reach statistical significance in this study. These results emphasize the crucial role of TNF-α in driving adipocytokine dysregulation, which likely contributes to the pro-inflammatory state observed in both AT and psoriatic skin [13]. The interplay between adipocytokines and psoriasis-signature cytokines underscores the potential for these mediators to serve as both biomarkers and therapeutic targets. Strategies aimed at restoring adipocytokine balance could break the vicious cycle linking AT inflammation and psoriasis [14].

These observations are consistent with previous studies showing that TNF-α acts as a key driver of AT inflammation, inducing a pro-inflammatory phenotype and upregulating chemokines such as MCP-1 and IL-6 [15,16]. The synergistic effects observed with the combination of IL-17 and TNF-α are particularly intriguing, as these cytokines are known to cooperate in amplifying immune responses in psoriasis [17]. This additive effect is likely mediated by their ability to act on distinct yet overlapping signaling pathways in AT, including NF-κB and STAT3, both of which are involved in inflammation and metabolic regulation. The downstream effects of cytokine-induced AT dysregulation were evident in the ex vivo skin organ culture experiments, where supernatants from cytokine-stimulated AT triggered significant upregulation of key inflammatory mediators in healthy skin fragments, including IL-6, IL-8, IL-23 and IL-36γ. These mediators are known to play central roles in psoriatic inflammation. For instance, IL-23 is a crucial driver of Th17 differentiation and the production of IL-17 itself, creating a positive feedback loop that perpetuates chronic inflammation [15,18]. Similarly, IL-36γ has been shown to amplify keratinocyte activation and pro-inflammatory responses in psoriatic skin [19]. Our data also reveal a marked disruption in the balance of adipokines following cytokine stimulation of AT. IL-17 significantly reduced adiponectin levels, while TNF-α, alone or in combination with IL-17, increased resistin secretion. These findings are supported by previous studies reporting reduced adiponectin and increased resistin levels in obese and psoriatic patients. Adiponectin, an anti-inflammatory adipokine, has been shown to inhibit IL-17 production by T cells [20]. Its downregulation in the presence of IL-17 could therefore perpetuate a vicious cycle of inflammation. Conversely, resistin and leptin, which were modestly upregulated in this study, are known to promote Th1 and Th17 immune responses and have been implicated in systemic inflammation and psoriasis severity [21,22]. These results provide a mechanistic explanation for the clinical association between obesity and psoriasis, where AT inflammation exacerbates skin disease and, reciprocally, psoriatic inflammation impacts AT by promoting a pro-inflammatory state that disrupts its metabolic and secretory functions, thereby contributing to systemic inflammation and disease progression. Several studies have demonstrated that higher BMI and adiposity are risk factors for both the onset and severity of psoriasis [23,24]. Moreover, obesity has been shown to impair the efficacy of psoriasis treatments, particularly fixed-dose biologics like TNF inhibitors, likely due to altered pharmacokinetics and increased systemic inflammation [9]. By elucidating the molecular crosstalk between AT and psoriatic skin, our findings highlight the importance of targeting this axis in therapeutic strategies. From a therapeutic perspective, interventions aimed at modulating AT inflammation could benefit psoriasis patients. Weight loss interventions, for example, have been shown to reduce systemic inflammation and improve psoriasis severity [25]. Targeting specific pathways, such as IL-17 or TNF-α signaling, with biologic therapies may not only alleviate skin symptoms but also attenuate AT inflammation and associated comorbidities. Furthermore, the restoration of adipokine balance, either pharmacologically or through lifestyle interventions, represents a potential avenue for mitigating the systemic effects of this bidirectional relationship. These findings underscore the importance of addressing AT inflammation in psoriasis management. Clinically, this suggests that interventions targeting AT inflammation, such as weight reduction strategies or therapies that restore adipokine balance, could enhance treatment outcomes. Moreover, the observed pro-inflammatory reprogramming of AT by psoriasis-signature cytokines may help explain the reduced efficacy of biologic therapies in obese patients, emphasizing the need for tailored therapeutic approaches in this subgroup. By integrating metabolic modulation with established treatments like TNF-α or IL-17 inhibitors, clinicians may break the vicious cycle between AT and psoriatic inflammation, ultimately improving both skin and systemic health outcomes.

## 4. Material and Methods

### 4.1. In Vitro AT Model

**Figure 4:**
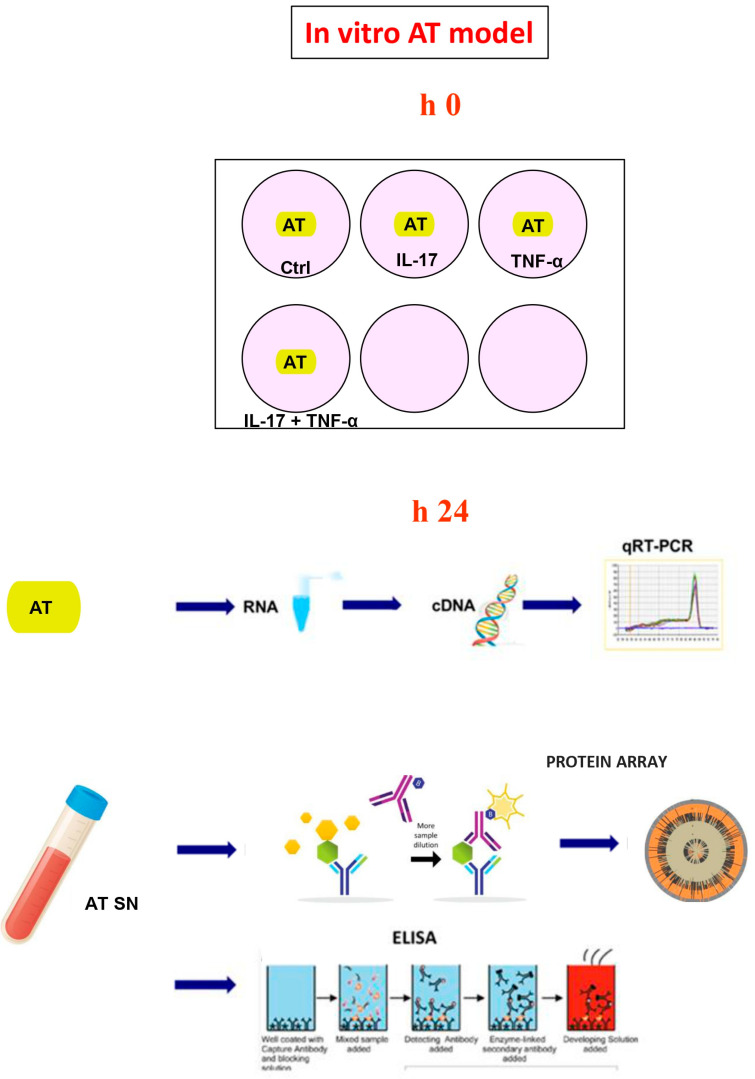
Illustration of AT in vitro model.

An in vitro experimental model reflecting the potential influence of psoriasis skin inflammation on AT was made (Figure 4). In brief, subcutaneous AT fragments, obtained from 7 healthy normal to overweight women undergoing mammal surgery for cosmetic reasons, were placed in culture. All study participants provided informed consent for the use of their tissues in this study. Sized fragments (500 mg) freely floating in 16 mL serum-free Medium 199 (GIBCO, Grand Island, NY, USA) without phenol red in 50 mL plastic tubes were cultivated in a humidified incubator and maintained at 37 °C and at an atmosphere of 5% CO_2_. Medium 199 was supplemented with 25 mM Hepes (Sigma-Aldrich, St. Louis, MO, USA), 5% bovine albumin (Sigma-Aldrich, St. Louis, MO, USA) and 1 nM insulin (Novo Nordisk, Bagsværd, Denmark). Subsequently, AT was stimulated with recombinant human (rh)-IL-17 (R&D System, Minneapolis, MN, USA; 200 ng/mL), TNF-α (Sigma-Aldrich, St. Louis, MO, USA; 10 ng/mL) or with the combination of both cytokines. For each tested variable, AT was incubated in triplicate, and the medium was collected after 24 h. AT was immediately frozen in liquid nitrogen and kept at −80 °C until the time of RNA isolation.

### 4.2. Protein Array

The latter conditioned medium was analyzed through protein array (Raybiotech, Norcross, GA, USA) which utilizes the sandwich immunoassay principle, wherein a panel of captured antibodies is printed on a nitrocellulose membrane solid support. The array membranes are processed through a chemiluminescent readout and signals are then visualized on X-ray film or digital image, allowing for densitometry data collection and the calculation of fold changes for each detected protein.

### 4.3. ELISA

In detail, the concentration of adiponectin was measured as previously described [26]. Leptin concentration was evaluated using the commercial Human Leptin (LEP) ELISA Kit according to the manufacturer’s instructions (Elabscience, Kampenhout, Belgium). Resistin amount was detected using a commercial kit according to the manufacturer’s instructions (Invitrogen, MA, USA). Each sample was assayed three times and in triplicate.

### 4.4. Ex Vivo Healthy Skin Organ Culture

**Figure 5:**
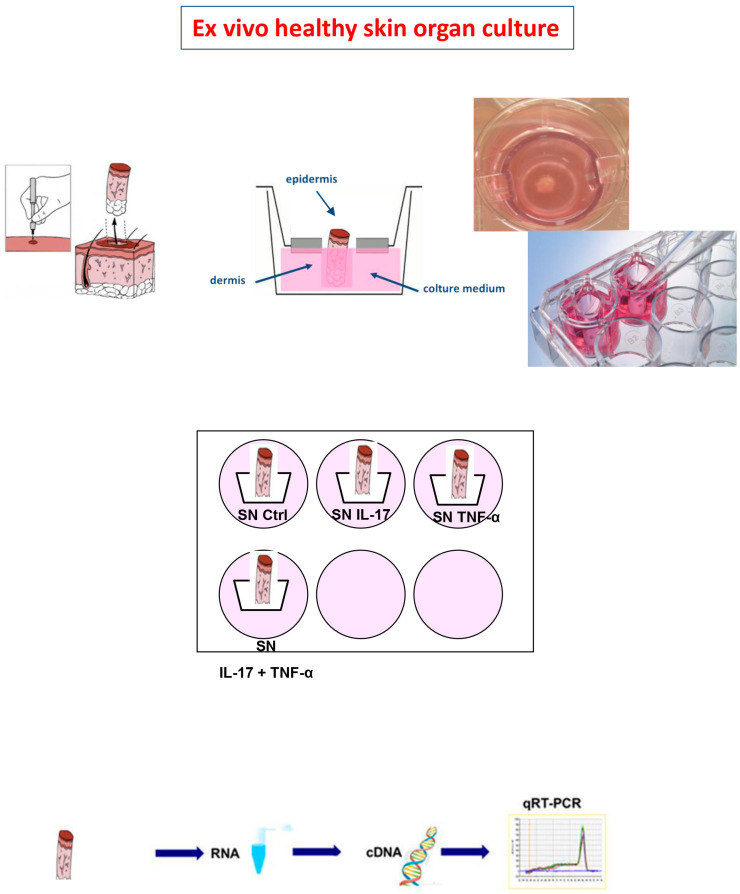
Illustration of ex vivo healthy skin organ culture.

In order to more deeply analyze the possible effects of inflamed AT on the skin, we performed ex vivo healthy skin organ culture (OC) assays with the collected supernatants of the above-mentioned AT experiments [27]. In brief, supernatants of AT previously stimulated with IL-17 (200 ng/mL), TNF-α (10 ng/mL) and the combination of these 2 cytokines were collected at 24 h and used to incubate the corresponding derived healthy skin OCs for 30 h (Figure 5). In detail, skin specimens were obtained from healthy normal to overweight women undergoing mammal surgery for cosmetic reasons. Skin fragments of 3 mm in diameter were cultured as follows: A hole was punched in a transwell filter (pore size 1 lm; Beckton Dickinson Labware, Franklin Lakes, NJ, USA). The skin specimen was inserted into the hole, and the filter containing the specimen was placed in a 12-well culture plate (Beckton Dickinson Labware) containing 1 mL of culture medium. In this system, the epidermis faces upwards at the liquid–air interface, whereas the dermis is suspended in the culture medium. The medium was composed of 500 µL of Dulbecco’s modified Eagle’s medium (DMEM; GIBCO, Grand Island, NY, USA) made up of 10% fetal bovine serum (FBS; GIBCO, Grand Island, NY, USA), 2 mM L-glutamine (GIBCO, Grand Island, NY, USA) and antibiotics (100 IU/mL penicillin G and 100 lg/mL streptomycin; (GIBCO, Grand Island, NY, USA) plus 500 µL of Medium 199 (GIBCO, Grand Island, NY, USA) supplemented with 25 mM Hepes (Sigma-Aldrich, St. Louis, MO, USA), 5% bovine albumin (Sigma-Aldrich, St. Louis, MO, USA) and 1 nM insulin (Novo Nordisk, Bagsværd, Denmark) that had been previously incubated with AT fragments for 24 h. The skin was immediately frozen in liquid nitrogen and kept at −80 °C until the time of RNA isolation.

### 4.5. RT-PCR

RNA was extracted from AT as well as skin OCs (RNeasy Mini Protocol; Qiagen) and cDNA was prepared (Transcriptor High Fidelity cDNA Synthesis; Roche; Indianapolis, IN, USA) according to the manufacturer’s instructions. RT-qPCR (LightCycler; Roche, Indianapolis, IN, USA) was used to analyze the levels of expression of 18S, Il-6, IL-8, IL-23, IL-36γ and IL-10. Relative mRNA levels were determined by using the comparative threshold cycle method 2-∆∆cq [28], and their expression was normalized to the expression of 18S mRNA, as previously reported [29]. PCR primers were designed based on published sequences, and their specificity was verified with a BLAST alignment search. To confirm the amplification of the expected size fragment, amplification products were characterized by agarose gel electrophoresis. Melting curve analysis was carried out after completion to confirm the presence of single amplified species.

### 4.6. Statistical Analysis

Each experiment was performed in triplicate and was repeated three times. Statistical analyses were performed using GraphPad Prism 6.0 (GraphPad Software Inc., La Jolla, CA, USA). Data that passed the normality test were analyzed with a two-tailed *t*-test; otherwise, the nonparametric Mann–Whitney test was used to calculate statistical differences. Values of *p* < 0.05 were considered significant. The data are expressed as means ± the standard deviation (SD).

## 5. Conclusions

In conclusion, this study demonstrates that IL-17 and particularly TNF-α reprogram AT into a pro-inflammatory state, disrupting its metabolic and secretory functions. The resulting AT-derived mediators amplify psoriatic skin inflammation, establishing a vicious cycle that links obesity and psoriasis. Understanding the molecular underpinnings of this crosstalk could pave the way for integrated treatment strategies targeting both AT and psoriatic skin, ultimately improving outcomes for patients with these inter-related conditions. Further research is warranted to explore these interactions in vivo and to evaluate targeted therapeutic approaches.

## Data Availability

The original contributions presented in the study are included in the article, further inquiries can be directed to the corresponding authors.
